# Choroidal thickness measurement by enhanced depth imaging and swept-source optical coherence tomography in central serous chorioretinopathy

**DOI:** 10.1186/1471-2415-14-145

**Published:** 2014-11-25

**Authors:** Ferdiriva Hamzah, Ari Shinojima, Ryusaburo Mori, Mitsuko Yuzawa

**Affiliations:** Department of Ophthalmology, School of Medicine, Surugadai Hospital of Nihon University, 1-8-13 Surugadai, Kanda, Chiyodaku, Tokyo, 101-8309 Japan; Jakarta Eye Center, Jakarta, Indonesia

## Abstract

**Background:**

We evaluated subfoveal choroidal thickness measured with two different forms of optical coherence tomography (OCT), enhanced-depth imaging (EDI) and swept-source (SS) OCT, in central serous chorioretinopathy (CSC).

**Methods:**

Fifty-six eyes of 48 patients diagnosed with acute or chronic CSC, were studied prospectively. Subfoveal choroidal thickness was measured as the distance between the outer border of the retinal pigment epithelium-Bruch’s membrane complex, and the chorioscleral border under the fovea. Subfoveal choroidal thickness was measured using EDI-OCT and SS-OCT. We also measured serous retinal detachment (SRD) only with SS-OCT. The Pearson correlation coefficient was used to assess the correlation between subfoveal choroidal thickness values determined by the two different OCT modalities.

**Results:**

The mean patient age was 52 ± 13 years (range, 32–82 years). Among the 56 eyes, 21 had acute CSC and 35 had chronic CSC. Subfoveal choroidal thickness measured with EDI-OCT was 336.6 ± 91.6 μm in acute and 388.0 ± 103.4 μm in chronic CSC. With SS-OCT, the thickness in acute CSC was 332.0 ± 96.7 μm and that in chronic CSC was 392.6 ± 101.3 μm. Acute CSC (p <0.001, correlation coefficient; r = 0.99) and chronic CSC (p <0.001, correlation coefficient; r = 0.97) values obtained with the two different OCT modalities correlated significantly. Among the 56 eyes, 43 (19 eyes with acute and 24 with chronic CSC) were evaluable for SRD height by SS-OCT. The mean SRD height was 128.9 ± 83.6 μm in acute cases and 96.3 ± 62.0 μm in chronic cases.

**Conclusions:**

Subfoveal choroidal thickness obtained with two different OCT modalities correlated significantly.

## Background

Choroidal abnormalities are associated with the pathogenesis of various disorders affecting the retina including central serous chorioretinopathy (CSC) [1, 2].

In CSC patients, the choroid is thicker than in normal eyes. Choroidal vascular hyper-permeability is frequently seen in eyes with CSC. This raises hydrostatic pressure in the choroid and thereby increases choroidal thickness [3, 4].

In recent years, detecting the choroid using spectral-domain optical coherence tomography (SD-OCT) has often been difficult, because the retinal pigment epithelium (RPE) hinders the penetration process. This is because the wavelength of the light source is occasionally not long enough to penetrate into the choroid. Conventional OCT employs a wavelength of about 800 nm, while the wavelengths capable of clear choroidal imaging are reportedly in the 1,060 nm range [5–7].

Enhanced depth imaging (EDI) OCT is a recent modification of the standard technique. This novel modality shows the cross-sectional structure and thickness of the choroid using commercial SD-OCTs. Basically, the EDI-OCT modality places the objective lens of the SD-OCT closer to the eye, such that the light backscattered from the choroid is closer to the zero-delay and sensitivity is thereby enhanced. Therefore, this modality produces better imaging of the choroid [8, 9].

However, even though EDI-OCT can enhance the sensitivity of the choroid, light scattering by the RPE and choroid remains a problem. In this event, the chorioscleral border cannot be detected, especially when a subject happens to have a particularly thick choroid. Measurable choroidal thickness has been reported in 95.8% of the eyes examined by Heidelberg EDI-OCT, while thickness cannot be determined in the remainder due to poor image quality obscuring the outer boundary of the choroid [10]. Yamashita et al. reported that choroidal thickness measurements were unsuccessful in some healthy eyes examined (4 out of 43 eyes) because the subjects had choroidal thicknesses exceeding 500 μm, making it impossible to clearly see the outer boundary of the choroid [11].

Topcon Deep Range Imaging OCT is another new technique designed to produce better imaging of the choroid. It has a scanning speed of 100,000 A-scans/second and is based on Swept-Source (SS) technology. It utilizes a wavelength of 1,050 nm and thereby provides deeper penetration into the choroid with higher image quality. The longer the wavelength of the light, the less is the scattering reflection of the RPE, thus enabling deep and full-thickness choroidal imaging even up to the scleral surface. SS-OCT showed a clearly defined, measurable posterior portion of the choroid [5–7, 12].

OCT has now been proven to be an effective non-invasive tool for evaluating and detecting choroidal changes in pathological states [13–15]. It is also important to evaluate and compare the measurements of choroidal thickness obtained using different instruments on subjects with pathological conditions. Moreover, SS-OCT can be used in addition to choroidal imaging in clinical practice [7, 13].

## Methods

Between November 2012 and September 2013, we studied 56 eyes of 48 CSC patients (41 men, 49 eyes; 7 women, 7 eyes) at Surugadai Nihon University Hospital, Tokyo, Japan. This study was approved by the institutional review board of Surugadai Nihon University Hospital and written informed concent was obtained from all patients. All subjects underwent a comprehensive ophthalmic examination, that included a thorough ocular examination using an indirect ophthalmoscope and slit-lamp biomicroscope with a contact lens, including an autorefractometer, best-corrected visual acuity (BCVA) measurement with the Landolt C eye chart, color fundus photography (TRC, 50IX/Imagenet, Topcon, Tokyo, Japan), intraocular pressure measurement, slit-lamp examination, dilated funduscopy, fluorescein angiography (FA), indocyanine green angiography, EDI-OCT and SS-OCT. CSC was objectively diagnosed based on the angiographic findings. If both eyes met the inclusion criteria, then the subfoveal choroidal thickness was measured regardless of whether the case was unilateral or bilateral, and the value was included in this study. Acute CSC was defined as detachment of the neurosensory retina which became symptomatic within 6 months. Chronic CSC was defined as detachment of the neurosensory retina in terms of symptoms, which persisted for at least 6 months. All subjects were imaged with Heidelberg Spectralis (Heidelberg Engineering Inc., Heidelberg, Germany) using the EDI-OCT technique and Topcon Deep Range Imaging (DRI) SS-OCT (Topcon Corp, Tokyo, Japan) on the same day around noon to avoid diurnal variations [16]. A horizontal section through the fovea was obtained for measurement. The scan was performed with each instrument using a single horizontal scan line. The length of the scan line was 12 mm in SS-OCT, 6 mm in EDI-OCT. There were 100,000 A-scans/second per line in SS-OCT, and 40,000 A-scans/second per line in EDI-OCT. Both instruments were equipped with an eye-tracking modality.

The subfoveal choroidal thickness was measured as the distance between the outer border of the RPE-Bruch’s membrane complex and the chorioscleral border under the fovea. It was measured manually using the caliper tool. If the line was blurred at the chorioscleral interface, the center of the traceable line was measured. We measured serous retinal detachment (SRD) only with SS-OCT, from the outer surface of the neurosensory retina and the RPE. Cases with a myopic refractive error exceeding 6.0 Diopters, a history of intraocular surgery, photodynamic therapy, other intraocular diseases including retinal dystrophies, uveitis, macular hole, epiretinal membrane, and/or macular edema not associated with CSC, were excluded from this study. Patients who had undergone focal laser photocoagulation were included in the study [3, 4].

All data were expressed as means ± standard deviation (SD). The Pearson correlation coefficient was used to assess the correlation between subfoveal choroidal thickness values determined by the two different OCT modalities. Statistical analysis was performed using SPSS software (Version 21.0 for Windows, IBM-SPSS, Japan).

## Results

**Figure 1:**
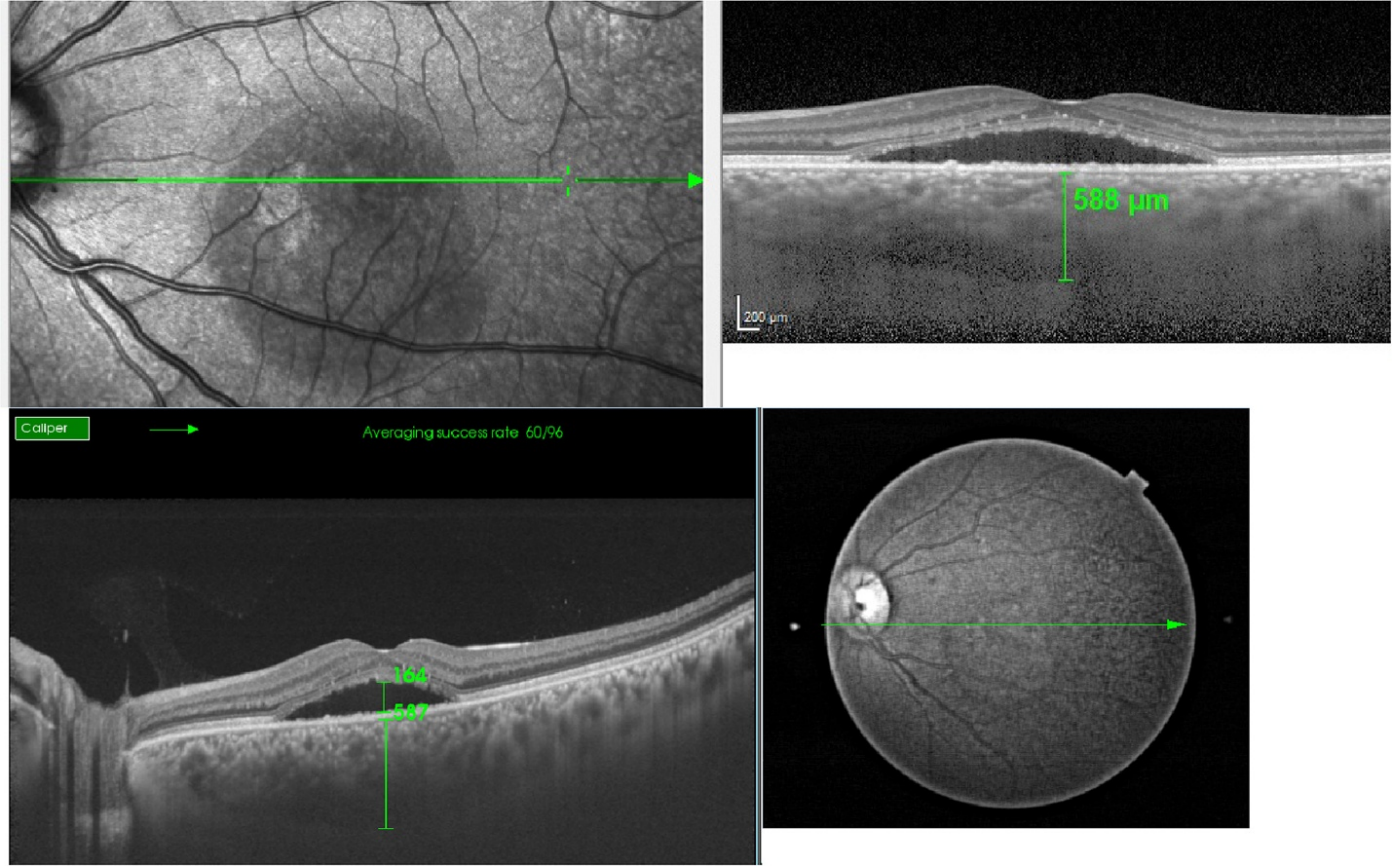
**Representative images obtained with two different forms of optical coherence tomography.** Chronic unilateral central serous chorioretinopathy, in the left eye, with onset 12 months prior to study enrollment. Enhanced depth imaging optical coherence tomography (EDI-OCT) (Top). Swept-source OCT (SS-OCT) image (Bottom). Both images were obtained on the same day. The subfoveal choroidal thickness was measured as the distance between the outer border of the retinal pigment epithelium-Bruch’s membrane complex and the chorioscleral border under the fovea. Subfoveal choroidal thickness was 588 μm in EDI-OCT (Top), and 587 μm in SS-OCT (Bottom). Serous retinal detachment was measured between the outer surface of the neurosensory retina and the retinal pigment epithelium, and the height was 164 μm in SS-OCT (Bottom).

**Figure 2:**
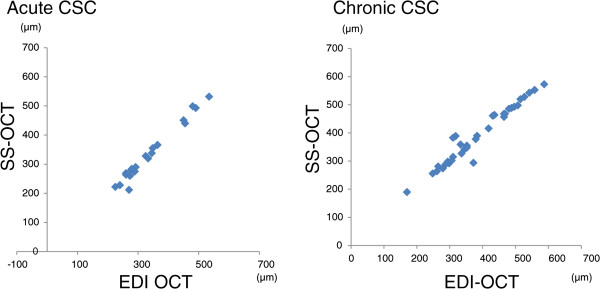
**Correlation between choroidal thickness values measured with two different forms of optical coherence tomography.** Both figures show the linear correlation between enhanced depth imaging optical coherence tomography (EDI-OCT) and swept-source OCT. Acute central serous chorioretinopathy (CSC) (n = 21, correlation coefficient; r = 0.99; p < 0.001) (Left figure). Chronic CSC (n = 35, correlation coefficient; r = 0.97; p < 0.001) (Right figure).

The mean age of the patients was 52 ± 13 years (range, 32–82 years). The mean refractive error was -0.1 ± 1.9 Diopters (mean ± SD, range, -5.5-3.75 Diopters). Among the 56 eyes, 21 had acute CSC and 35 had chronic CSC. The mean BCVA was 1.1 ± 0.4 decimals (mean ± SD, range, 0.2-1.5). Mean intraocular pressure was 15.4 ± 2.7 mmHg (mean ± SD, range, 9–21 mmHg). The duration of acute CSC from the onset was 7.4 ± 6.6 weeks (mean ± SD, range, 1–20 weeks), and the duration of chronic CSC from the onset was 62.1 ± 51.5 weeks (mean ± SD, range, 26–260 weeks). Good quality B-scan images from EDI- and SS-OCT (Figure 1) were obtained for all eyes. With SS-OCT, the mean SRD heights in acute and chronic CSC cases were 128.9 ± 83.6 μm and 96.3 ± 62.0 μm, respectively.Subfoveal choroidal thickness in acute CSC measured with EDI-OCT was 336.6 ± 91.6 μm (mean ± SD, range, 225–534 μm) and that in chronic CSC was 388.0 ± 103.4 μm (mean ± SD, range, 170–587 μm). With SS-OCT, the thickness in acute CSC was 332.0 ± 96.7 μm (mean ± SD, range, 212–532 μm) and that in chronic CSC was 392.6 ± 101.3 μm (mean ± SD, range, 190–573 μm). Choroidal thickness values obtained with the two different OCT modalities showed a significant correlation. Acute CSC (p < 0.001, correlation coefficient; r = 0.99) and chronic CSC (p < 0.001, correlation coefficient; r = 0.97) values are presented in Figure 2.

## Discussion

EDI is a recently standardized technique for imaging the choroid more clearly using commercial SD-OCT [17–20]. Yamashita et al. reported that 90.7% of subjects had measurable choroidal thickness when examined using three different SD-OCT modalities, including EDI-OCT [11].

Topcon DRI is another new form of OCT that provides higher penetration enabling deep choroidal imaging. Moreno et al. reported 100% of the definite chorioscleral junction to be visualized with SS-OCT (DRI) [5]. The SS-OCT facilitated visualization for reliable thickness measurements. The EDI-OCT occasionally requires the contrast to be set in order to trace the chorioscleral borders clearly, especially in patients with a subfoveal choroidal thickness exceeding 500 μm. No eyes were excluded from our study due to poor-quality EDI-OCT images. Occasionally, it was necessary to change the image contrast, but this allowed us to detect the chorioscleral border in all eyes, even those with a maximum thickness of 587 μm on EDI-OCT, and a maximum thickness of 573 μm on SS-OCT.

Normal subfoveal choroidal thickness varies widely among reports [7, 8, 21]. Ikuno et al. [7] found that the choroidal thickness values in healthy eyes correlated well between the 1,060 nm and 870 nm OCT instruments, but their report did not include eyes with pathological conditions.

In our study, even in symptomatic eyes with subretinal fluid, the choroidal thickness values obtained with two different OCT modalities showed a significant correlation. This was true for both acute CSC (p <0.001, correlation coefficient; r = 0.99) and chronic CSC (p <0.001, correlation coefficient; r = 0.97).

Imamura et al. [9] reported the subfoveal choroidal thickness measured with EDI-OCT in CSC to be greater than that in normal eyes. Maruko et al. [4] reported choroidal thickness, as measured with EDI-OCT, to be 344 ± 112 μm in acute CSC and 360 ± 123 μm in chronic CSC. Ferrara et al. [22] reported choroidal thickness measured with SS-OCT to be 351 ± 84 μm in chronic CSC. Compared with normal eyes, subfoveal choroidal thickness was significantly increased in both the eyes with active CSC and in the unaffected fellow eyes (P < 0.001 in both groups) [23].

In this study, chronic CSC was also associated with a slightly thicker choroid than acute CSC. Increased hydrostatic pressure develops within the choroid of CSC patients and it is possible that the choroidal thickness is thereby altered.

Negi and Marmor demonstrated in their experimental studies that focal damage, such as that produced by photocoagulation, appears to facilitate water movement from, rather than into, the subretinal space [24]. RPE damage in chronic CSC and photocoagulation scar findings appear to correspond to window defects, especially those observed on FA. Chronic CSC is associated with extensive RPE damage in the macular area, suggesting slightly more rapid resorption than in acute CSC. Therefore, subretinal fluid presumably flows toward the choroid and might thereby cause choroidal thickening through accumulation of fluid. This may be one of the factors leading to a thicker choroid in chronic as opposed to acute CSC cases. Ferrara et al. [22] reported that En face SS-OCT imaging at the level of the choriocapillaris showed hypoechoic spots suggesting the presence of enlarged vessels at this level in 8 (53%) of the eyes in their study.

To summarize the characteristics of CSC in a large number of publications [25], the cut-off value for the height of SRD, acute vs chronic CSC cases, is 100 μm, i.e., acute CSC >100 μm. In our study as well, with SS-OCT, the mean SRD heights in acute and chronic CSC cases were 128.9 ± 83.6 μm and 96.3 ± 62.0 μm, respectively.

This study has limitations, including manual measurement of subfoveal choroidal thickness on both EDI-OCT and SS-OCT (DRI) and the small sample size. Automatic detection and segmentation systems are still lacking for the choroid.

## Conclusion

Measuring choroidal thickness has become a tool facilitating the diagnosis of CSC. EDI-OCT and SS-OCT are useful for evaluating subclinical choroidal abnormalities in CSC and this study confirmed a correlation between the values obtained with these two OCT modalities.
